# Early haematologic response to anifrolumab in SLE: results from the European multicentre EFFORT-SLE study

**DOI:** 10.1093/rheumatology/keag400

**Published:** 2026-08-11

**Authors:** Michela Gasparotto, Alessandra Bettiol, Ginevra De Marchi, Edoardo Biancalana, Giulia Duro, Filippo Fagni, Alice Horisberger, Joško Mitrović, Alessia Nano, Katarzyna Wawrzycka-Adamczyk, Myriam Reisch, Lea Salamon, Georg Schett, Ladislav Šenolt, Elena Silvestri, Jens Thiel, Paola Tomietto, Krzysztof Wójcik, Jakub Závada, Luca Quartuccio, Giacomo Emmi

**Affiliations:** Department of Medical, Surgery and Health Sciences, University of Trieste, Italy and Clinical Medicine and Rheumatology Unit, Cattinara University Hospital, Trieste, Italy; Department of Experimental and Clinical Biomedical Sciences “Mario Serio”, University of Florence, Florence, Italy; Rheumatology Division, Department of Medicine, University of Udine and Azienda Sanitaria Universitaria Friuli Centrale, Udine, Italy; Department of Experimental and Clinical Medicine, University of Florence, Florence, Italy; Department of Medical, Surgery and Health Sciences, University of Trieste, Italy and Clinical Medicine and Rheumatology Unit, Cattinara University Hospital, Trieste, Italy; Department of Medicine 3—Rheumatology and Immunology, Friedrich-Alexander-Universität (FAU) Erlangen-Nürnberg and Universitaetsklinikum Erlangen, Erlangen, Germany; Centre Hospitalier Universitaire Vaudois, Immunology and Allergy Service, University of Lausanne, Lausanne, Switzerland; Division of Clinical Immunology, Allergology and Rheumatology, Department of Internal Medicine, Dubrava University Hospital, and University of Zagreb, School of Medicine, Faculty of Pharmacy and Biochemistry, Zagreb, Croatia; Rheumatology Division, Department of Medicine, University of Udine and Azienda Sanitaria Universitaria Friuli Centrale, Udine, Italy; Center for the Development of Therapies for Civilization and Age Related Diseases, Jagiellonian University Medical College, Cracow, Poland; Division of Rheumatology and Clinical Immunology, Medical University of Graz, Graz, Austria; Division of Clinical Immunology, Allergology and Rheumatology, Department of Internal Medicine, Dubrava University Hospital, and University of Zagreb, School of Medicine, Faculty of Pharmacy and Biochemistry, Zagreb, Croatia; Department of Medicine 3—Rheumatology and Immunology, Friedrich-Alexander-Universität (FAU) Erlangen-Nürnberg and Universitaetsklinikum Erlangen, Erlangen, Germany; Institute of Rheumatology and First Faculty of Medicine, Charles University, Prague, Czech Republic; Department of Experimental and Clinical Medicine, University of Florence, Florence, Italy; Division of Rheumatology and Clinical Immunology, Medical University of Graz, Graz, Austria; Department of Medical, Surgery and Health Sciences, University of Trieste, Italy and Clinical Medicine and Rheumatology Unit, Cattinara University Hospital, Trieste, Italy; 2nd Department of Internal Medicine, Faculty of Medicine, Jagiellonian University Medical College, Cracow, Poland; Institute of Rheumatology and First Faculty of Medicine, Charles University, Prague, Czech Republic; Rheumatology Division, Department of Medicine, University of Udine and Azienda Sanitaria Universitaria Friuli Centrale, Udine, Italy; Department of Medical, Surgery and Health Sciences, University of Trieste, Italy and Clinical Medicine and Rheumatology Unit, Cattinara University Hospital, Trieste, Italy; Centre Hospitalier Universitaire Vaudois, Immunology and Allergy Service, University of Lausanne, Lausanne, Switzerland; Centre for Inflammatory Diseases, Monash University Department of Medicine, Monash Medical Centre, Melbourne, Australia

**Keywords:** SLE, anifrolumab, cytopenia, anaemia, lymphopenia, thrombocytopenia, IFN, observational study, treatment outcome, autoimmune haemolytic anaemia

## Abstract

**Objectives:**

To evaluate the efficacy and safety of anifrolumab (ANI) for the haematologic manifestations of SLE in routine clinical practice.

**Methods:**

In this retrospective multicentre European study, adult patients with SLE presenting with ≥1 disease-related haematologic abnormality at ANI initiation were included. Clinical and laboratory data were collected at baseline and at 3, 6, 12, 18 and 24 months. Complete haematologic response (CHR) was defined as normalization of all baseline abnormalities, whereas partial haematologic response (PHR) was defined as normalization of at least one affected lineage.

**Results:**

Forty-seven patients from seven European countries were included (91.5% female). ANI was specifically initiated for haematologic involvement in 21/47 (44.7%), whereas 26/47 (55.3%) started treatment for other SLE manifestations but had ≥1 haematologic abnormality at baseline. CHR was achieved in 8/43 (18.6%) patients at 3 months and cumulatively in 14/46 (30.4%) within 6 months (overall 17/47, 36.2%), with comparable rates regardless of treatment indication. PHR occurred in 17/43 (39.5%) at 3 months and in 11/37 (29.7%) at 6 months. Haemoglobin, leucocyte and lymphocyte counts significantly improved by month 3, whereas platelet counts showed a non-significant increase. Higher baseline CRP, ESR and SLE-DAS were associated with the lack of CHR. Twelve patients (25.5%) discontinued ANI, and 17 (36.2%) experienced adverse events without discontinuation.

**Conclusion:**

ANI was associated with early haematologic improvement, with one-third of patients achieving complete normalization under a stringent definition of remission. Responses involved multiple haematologic lineages and were less frequent in patients with higher inflammatory activity, although confirmation in broader populations is warranted.

Rheumatology key messages

- Approximately one-third of SLE patients achieved complete haematologic normalization within 6 months of anifrolumab.
- Haematologic improvement involved multiple lineages, particularly haemoglobin, leucocytes and lymphocytes.
- Higher baseline inflammatory activity was associated with a lower probability of haematologic response.

## Introduction

SLE is a chronic multisystem autoimmune disease characterized by autoantibody production, immune complex deposition and persistent inflammation. Its clinical course is heterogeneous, frequently involving skin, joints, kidneys and the haematologic system. Haematologic manifestations, including leukopenia, lymphopenia, thrombocytopenia, autoimmune haemolytic anaemia (AHA) and, in severe cases, macrophage activation syndrome (MAS), are common and contribute substantially to disease activity and morbidity [1]. Haematologic involvement reflects a combination of immune-mediated peripheral destruction, complement-mediated clearance and bone marrow dysregulation driven by chronic inflammatory signalling [2]. Type I IFNs, particularly IFN-α, play a central role in SLE pathogenesis and are implicated in the development of cytopenias [3]. IFN-I signalling interferes with haematopoiesis through inhibition of progenitor proliferation, disruption of differentiation pathways and alteration of the marrow microenvironment, impairing erythropoiesis, lymphoid output and platelet production. IFN pathway activation has also been associated with severe SLE-related complications such as MAS [4]. Together with peripheral immune mechanisms, these effects may contribute to cytopenias in a subset of patients [5].

Anifrolumab (ANI), a fully human monoclonal antibody targeting the type I IFN receptor subunit 1 (IFNAR1), inhibits signalling from all type I IFNs and is approved for the treatment of moderate-to-severe SLE. Current EULAR recommendations recognize ANI as a therapeutic option in patients with persistently active disease despite standard therapy [6]. In the phase III TULIP-1 and TULIP-2 trials, ANI reduced overall disease activity and improved mucocutaneous and musculoskeletal manifestations. Post-hoc analyses suggested benefit also in the haematologic domain; however, patients with prominent cytopenias were underrepresented and specific data remain limited [7].

In light of this biological rationale and the limited data on haematologic outcomes, the EFFORT-SLE (Early Haematologic Response to Anifrolumab in Systemic Lupus Erythematosus) study was designed to evaluate the efficacy and safety of ANI in patients with SLE with baseline cytopenias in routine clinical practice across Europe.

## Methods

### Study population and definitions

In this retrospective multicentre study, we included adult patients with SLE fulfilling the 2019 ACR/EULAR classification criteria who were treated with ANI at nine European referral centres across seven countries (Austria, Croatia, Czech Republic, Germany, Italy, Poland and Switzerland). ANI was prescribed according to routine clinical practice and national prescribing recommendations.

Baseline haematologic involvement was defined by the presence of ≥1 of the following: haemoglobin <12 g/dl; platelets <150 000/mm³; leucocytes <4000/mm³; lymphocytes <1000/mm³; neutrophils <1000/mm³; AHA, defined as anaemia with laboratory evidence of haemolysis, including elevated lactate dehydrogenase, indirect hyperbilirubinaemia and/or reduced haptoglobin, in association with a positive direct antiglobulin test; or MAS, defined according to the HLH-2004 diagnostic criteria.

Patients were excluded if cytopenias were attributable to alternative causes, if transfusion support or bone marrow growth factors were administered during ANI therapy, or if glucocorticoid dose escalation or initiation of additional immunosuppressive therapy occurred within 3 months before or the month following ANI initiation.

For each patient, demographic and clinical characteristics, laboratory data and disease activity indices were collected at baseline and at 3, 6, 12, 18 and 24 months after treatment initiation. Disease activity was assessed using total SLEDAI-2K, the haematologic items of SLEDAI-2K and SLE-DAS. Complete haematologic response (CHR) was defined as normalization of all baseline haematologic abnormalities; partial haematologic response (PHR) was defined as normalization of at least one haematologic alteration. Cytopenia normalization was defined according to standard clinical laboratory reference thresholds for each parameter.

### Statistical analyses

Categorical variables are presented as frequencies and percentages, and continuous variables as mean (standard deviation [SD]) or median (interquartile range [IQR]), according to data distribution. Baseline characteristics of responders and non-responders were compared using Fisher’s exact test for categorical variables and the exact Mann–Whitney test for continuous variables. The proportion of patients achieving CHR or PHR was compared in patients starting ANI for haematologic vs other manifestations, using Fisher’s exact test. Longitudinal changes in haematologic parameters at a given follow-up timepoints as compared with the baseline were assessed using the Wilcoxon signed-rank test for paired data, with Bonferroni correction for multiple comparisons (significant if *P* < 0.01). Multivariable analysis was not feasible due to sample size. Longitudinal analyses were performed to evaluate the impact of time and the reason for treatment initiation on complete response rates, using a linear mixed-effects model. The model included time (treated as a categorical variable: 0, 6, 12, 18 and 24 months), the reason for treatment beginning and their interaction as fixed effects. To account for inter-individual variability, a random intercept was incorporated for each patient. Estimated marginal means with 95% confidence intervals (95% CI) were calculated to compare group trajectories over time. The significance level was set at *P* < 0.05.

Statistical analyses were performed using Stata SE version 18; graphs were generated with Prism 10.

The study was approved by the Area Vasta Centro Ethics Committee of Tuscany, Italy (Em. 2025-098 ID 23813_oss).

## Results

### Baseline characteristics

A total of 47 patients were included. Forty-three (91.5%) were female, and 38 (80.9%) were Caucasian. The median age at diagnosis was 31 years (IQR 21–44), and the median disease duration at ANI initiation was 10 years (IQR 4–18). None of the enrolled patients required glucocorticoid dose escalation or initiation of a new immunosuppressive therapy after ANI initiation.

At treatment initiation, mucocutaneous involvement was present in 66.0% of patients, articular activity in 57.4% and constitutional manifestations in 44.7%. ANI was started specifically for haematologic manifestations in 21 patients (44.7%), whereas 26 (55.3%) initiated treatment for other SLE manifestations but had ≥1 haematologic abnormality at baseline.

Regarding haematologic findings at baseline, lymphopenia was present in 31 patients (66.0%), anaemia in 23 (48.9%) and thrombocytopenia in 14 (29.8%). Six patients (12.8%) had active AHA and one fulfilled criteria for MAS. Follow-up data were complete for all patients at 3 months and were available for 38 (80.9%), 23 (48.9%), 9 (19.1%) and 7 (14.9%) patients at 6, 12, 18 and 24 months, respectively. The number of patients evaluable for haematologic response at each timepoint varied according to the availability of complete laboratory data.

Detailed baseline clinical, laboratory and immunological characteristics are reported in Table 1.

**Table 1:** Baseline demographic, clinical and laboratory characteristics of the study population.

Variable	*N* (%) or median (IQR), as appropriate
Demographic characteristics	
Female sex	43 (91.5)
Median age at diagnosis, years	31 (21–44)
Median disease duration before ANI, years	10 (4–18)
Caucasian ethnicity	38 (80.9)
Other ethnicity	9 (19.1)
Clinical manifestations at ANI initiation	
Mucocutaneous	31 (66.0)
Articular	27 (57.4)
Constitutional	21 (44.7)
CNS/PNS	6 (12.8)
Renal	3 (6.4)
Serositis	3 (6.4)
Gastrointestinal	1 (2.1)
Other	2 (4.3)
Haematologic abnormalities	
Lymphocytes <1000 cells/mm³	31 (66.0)
Haemoglobin <12 g/dl	23 (48.9)
Leucocytes <4000 cells/mm³	18 (38.3)
Platelets <150 000 cells/mm³	14 (29.8)
Haemolytic anaemia	6 (12.8)
Neutrophils <1000 cells/mm³	2 (4.3)
Macrophage activation syndrome	1 (2.1)
Serology	
ANA positive	47 (100.0)
Anti-dsDNA positive	30 (63.8)
Anti-Sm positive^a^	15 (55.7)
Antiphospholipid antibody positivity (any)	13 (27.7)
C3, mg/dl	79 (68–95)
C4, mg/dl	11 (7–17)

a Percentage calculated among tested patients. Continuous variables are reported as median (IQR).

ANI: anifrolumab; IQR: interquartile range; PNS: Peripheral Nervous System.

### Overall haematologic outcomes

CHR and PHR were assessed at each scheduled evaluation timepoint.

CHR was achieved in 8/43 patients (18.6%) at 3 months and in 14/46 (30.4%) within 6 months of treatment; overall, 17/47 patients (36.2%) achieved CHR at any time during follow-up. Considering any timepoint, CHR occurred in 8/21 patients (38.1%) who initiated ANI for haematologic involvement and in 9/26 (34.6%) who started treatment for other SLE manifestations. PHR was observed in 17/43 patients (39.5%) at 3 months, 11/37 (29.7%) at 6 months and 5/20 (25.0%) at 12 months. No additional PHR events were documented at subsequent evaluations. Three patients who had previously achieved PHR subsequently lost this status during follow-up.

CHR and PHR rates at scheduled visits and cumulative CHR rates according to the indication for ANI initiation are detailed in Supplementary Table S1. Active organ involvement considered as a reason for ANI initiation in patients who started ANI for haematologic involvement and in those who started it for other clinical manifestations is summarized in Supplementary Table S2.

### Longitudinal analysis of haematologic response

The linear mixed-effect model revealed a significant interaction between the reason for treatment initiation and the timepoint (overall Wald χ^2^, *P* < 0.001), indicating that CHR trajectories differed significantly between the two groups. Specifically, patients starting ANI for haematologic manifestations exhibited a more sustained and progressive improvement, reaching a higher and more durable CHR between 12 and 18 months. Conversely, patients starting ANI for other manifestations showed an early response, peaking at 6 months. The difference in trajectories between patients starting ANI for haematologic manifestations and those starting ANI for other manifestations was statistically significant at both 12 months (interaction coefficient: +0.34, *P* = 0.035) and 18 months (interaction coefficient: +0.52, *P* = 0.029). However, these results should be taken with caution, due to the limited number of patients with available data, particularly after 12 months of follow-up.

### Cytopenia-specific trajectories

**Figure 1:**
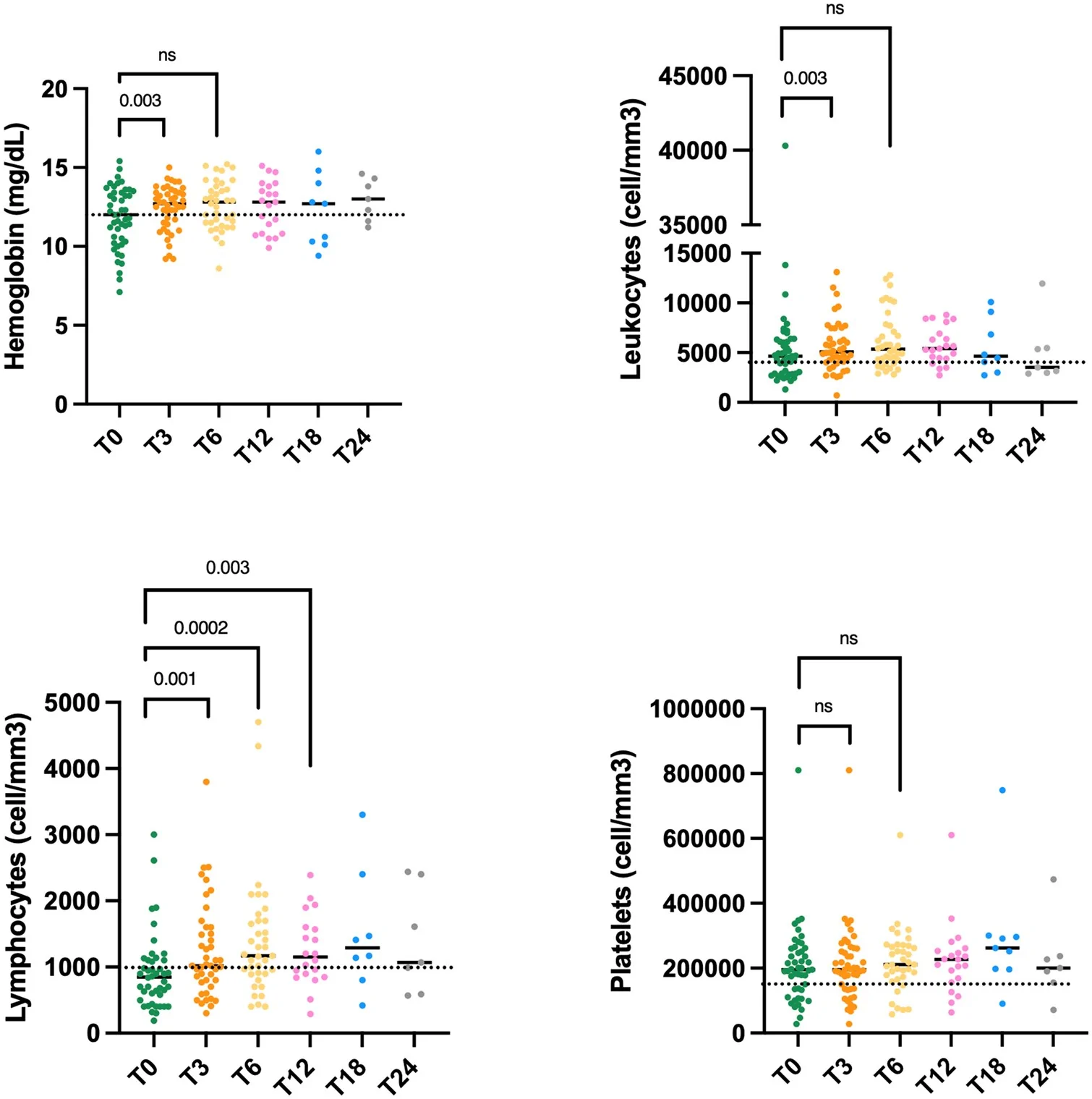
Longitudinal changes in haematologic parameters during ANI treatment. Individual patient values and median (IQR) are shown at each scheduled visit. Statistical comparisons refer to changes from baseline at 3, 6 and 12 months of follow-up. Dotted lines indicate the lower limit of the normal range for each haematologic parameter

Changes in individual haematologic parameters were evaluated longitudinally at 3, 6, 12, 18 and 24 months (Fig. 1).

Haemoglobin level (*P* = 0.003), leucocytes (*P* = 0.003) and lymphocyte counts (*P* = 0.001) significantly increased after 3 months, with lymphocytes levels keeping significantly higher compared with baseline also after 6 (*P* = 0.0002) and 12 months (*P* = 0.003).

Platelet counts showed an upward trend during follow-up; however, differences did not reach statistical significance at any scheduled timepoint.

Further analyses were performed considering longitudinal changes in haematologic parameters, only in patients presenting with an abnormal baseline value for the given parameter (Supplementary Fig. S1). Results confirm a significant increase in haemoglobin, leucocyte and lymphocyte values after 3 and 6 months of therapy, supporting haematologic improvement also in this subgroup of patients.

### Clinical and laboratory variables associated with CHR

Baseline characteristics were compared between patients who achieved CHR within 6 months and those who did not (Supplementary Table S3). In exploratory univariate analyses, CRP (*P* = 0.008), ESR (*P* < 0.001) and haematologic SLE-DAS (*P* = 0.033) differed significantly between responders and non-responders. CRP, ESR and haematologic SLE-DAS values were higher in non-responders. Considering specific subsets, among patients with AHA, four were non-responders, one reached the CHR after 12 months and one reached a PHR after 3 months; the case of the patient with MAS has been described elsewhere in detail [8]; lastly, the only patient with severe thrombocytopenia (<50 000 cell/mm^3^) displayed a partial response for haemoglobin, but not for platelet count.

No significant differences were observed for other clinical manifestations, complement levels, autoantibody profile or indication for ANI initiation.

### Safety

Infections were the most frequently reported adverse events and were predominantly mild, not requiring treatment discontinuation. Twelve patients (25.5%) discontinued ANI during follow-up. The most common reasons for discontinuation were inefficacy (4/12, 33.3%) and infections (3/12, 25.0%). Adverse events accounted for 2/12 (16.7%) discontinuations. The median time to discontinuation was 6 months (IQR 3.5–6.5).

No unexpected safety signals were observed. No patient discontinued treatment because of worsening haematologic parameters. Details on the safety profile are reported in Supplementary Table S4.

### Background therapy

Background therapy at baseline and at last follow-up is summarized in Supplementary Table S5.

Glucocorticoid use decreased over time, with the proportion of patients receiving glucocorticoids declining from 36/47 patients (76.6%) at baseline to 31/47 (66.0%) at last follow-up. The median daily prednisone dose reduced from 5 mg (IQR 2.5–10) to 2.5 mg (IQR 0–5). The use of conventional immunosuppressants and other biologic agents also reduced at last follow-up, whereas mycophenolate mofetil exposure remained unchanged.

## Discussion

Haematologic manifestations represent a frequent and clinically relevant component of SLE, ranging from mild laboratory abnormalities to severe, refractory and potentially life-threatening conditions [9]. Although the therapeutic armamentarium for SLE has expanded in recent years [6], real-world evidence supporting the efficacy of newer targeted therapies across specific organ domains remains limited, and dedicated data on haematologic outcomes under IFN pathway inhibition remain scarce.

To our knowledge, this is the largest real-world multicentre European cohort evaluating haematologic outcomes in patients with SLE treated with ANI, irrespective of baseline cytopenia severity. Previous evidence has been largely limited to case reports and small case series, including a single-country cohort of 10 patients mainly focused on severe or refractory haematologic phenotypes [8–12]. The inclusion of referral centres across multiple European countries enhances the generalizability of our findings across diverse healthcare settings and patient populations.

Approximately one-third of patients achieved CHR within 6 months, with a similar proportion attaining PHR during the same timeframe. CHR was intentionally defined stringently as normalization of all baseline haematologic abnormalities in order to provide a conservative estimate of haematologic remission and avoid overestimating clinically meaningful response. Under this definition, most responses occurred within the first months of therapy, identifying a clear early response window. This temporal pattern is consistent with the rapid effects of ANI observed in other disease domains, particularly mucocutaneous involvement, in both clinical trials and real-world studies [13–15]. Additional responses beyond 6 months were uncommon, suggesting that most haematologic improvement occurs early during treatment, although the stringent CHR definition may underestimate clinically meaningful partial improvements.

The magnitude of improvement differed across haematologic lineages, although responses occurred early across all parameters. Lymphocyte counts showed the most pronounced and sustained increase, remaining significantly higher than baseline up to 12 months. Haemoglobin and leucocyte counts increased significantly after 3 months and remained numerically higher thereafter, although statistical significance was not maintained, likely reflecting the limited number of patients at later timepoints. Platelet counts showed a non-significant upward trend, while patients with AHA demonstrated limited response, possibly reflecting distinct pathogenic mechanisms and a reduced contribution of IFN-driven pathways to autoantibody-mediated cytopenias. However, given the very small number of patients with AHA, MAS and severe thrombocytopenia included in our cohort, these observations should be considered exploratory and require confirmation in larger studies. Evidence on platelet response to ANI remains limited [12, 16] and larger cohorts are needed to clarify this aspect. Overall, the heterogeneous trajectories observed across cell lineages likely reflect the multifactorial pathogenesis of cytopenias in SLE [17].

Haematologic responses were observed both in patients initiating ANI for haematologic involvement and in those treated primarily for other disease manifestations. Although the study was not powered to detect subgroup differences, comparable response rates suggest that IFN-receptor blockade may confer haematologic benefit irrespective of the primary treatment indication. Exploratory longitudinal analyses suggested that the temporal pattern of response may differ according to the indication for ANI initiation, with a more sustained response observed among patients treated for haematologic manifestations. However, given the limited number of patients with long-term follow-up, these findings should be interpreted cautiously and require confirmation in larger cohorts.

Notably, patients achieving a CHR included those presenting only with baseline leukopenia or lymphopenia. Although mild forms of these abnormalities may not always require immediate therapeutic intervention, they remain relevant manifestations of disease activity, contribute to composite disease activity indices and have been associated with an increased risk of infection. Therefore, their inclusion provides a more comprehensive assessment of haematologic response in SLE.

In exploratory univariate analyses, higher baseline CRP and ESR levels, as well as higher haematologic SLE-DAS scores, were associated with a lower likelihood of achieving CHR within 6 months. These findings suggest that greater inflammatory burden and haematologic disease activity at baseline may reduce the probability of complete normalization, possibly reflecting partially IFN-independent inflammatory mechanisms. No significant associations were observed for complement levels, anti-dsDNA positivity, antiphospholipid antibodies or extra-haematologic manifestations. Given the limited sample size, these findings should be interpreted cautiously and require confirmation in larger cohorts.

During follow-up, 25.5% of patients discontinued ANI, mainly due to inefficacy or infections. Infections were the most frequently reported adverse events, although most did not require treatment discontinuation. The discontinuation rate appears higher than that reported in integrated analyses of phase II–III trials [18] and in preliminary real-world data [19], possibly reflecting greater disease complexity, prior treatment exposure and broader patient heterogeneity in routine practice. No discontinuations were attributed to worsening haematologic parameters.

Background therapy analysis showed a reduction in glucocorticoid use over time, both in the proportion of treated patients and in median daily dose, consistent with previously reported glucocorticoid-sparing effects of ANI [20]. The use of conventional immunosuppressants and other biologic agents also decreased, while mycophenolate mofetil exposure remained stable. Patients requiring transfusions or bone marrow growth factors during follow-up, as well as those undergoing glucocorticoid dose escalation or initiation of additional immunosuppressive therapy within 3 months before or the month following ANI initiation, were not eligible for inclusion, reducing the likelihood that haematologic improvement reflected concomitant treatment modifications, although these criteria may have selected a more clinically stable population.

This study has several limitations. Its retrospective design limits control of potential confounding factors. The relatively small sample size, particularly within specific cytopenia subgroups such as thrombocytopenia, restricts lineage-specific analyses. IFN gene-expression signatures were not available, preventing evaluation of the relationship between IFN pathway activation and haematologic response. In addition, follow-up beyond 6 months included limited patient numbers, particularly at 18–24 months, constraining interpretation of long-term outcomes.

Despite these limitations, our data provide clinically relevant insight into haematologic response to ANI in routine practice. Most responses occurred within the first 3–6 months of treatment, with approximately one-third of patients achieving complete haematologic normalization under a stringent definition requiring normalization of all affected lineages. These findings expand the limited real-world evidence on type I IFN receptor blockade in haematologic SLE and may help clinicians contextualize expected response patterns in clinical practice, although confirmation in broader and less selected populations is warranted.

## Supplementary Material

keag400_Supplementary_Data

## Data Availability

The datasets generated and analysed during the current study are not publicly available due to patient privacy and institutional data protection regulations but are available from the corresponding author on reasonable request, subject to approval by the participating centres and applicable ethical and legal requirements.
