# Supplementary material for: Early haematologic response to anifrolumab in SLE: results from the European multicentre EFFORT-SLE study
Source: Rheumatology (Oxford). 2026 Aug 11;65(9):keag400. doi: 10.1093/rheumatology/keag400 (PMC13544960; doi:10.1093/rheumatology/keag400)
Supplement: Supplementary file 1 [file keag400_supplementary_data.docx]

**Supplementary Table S1.** Achievement of hematologic response according to the indication for anifrolumab initiation

|  | Baseline | 3 months | 6 months | 12 months | 18 months | 24 months | Within 6 months | Any time |
| --- | --- | --- | --- | --- | --- | --- | --- | --- |
| Complete hematologic response | 0/47 | 8/43  (18.6%) | 10/37  (27.0%) | 7/20  (35.0%) | 3/8  (37.5%) | 2/7  (28.6%) | 14/46  (30.4%) | 17/47  (36.2%) |
| ANI started for hematologic manifestations | 0/21 | 3/20  (15.0%) | 3/14  (21.4%) | 5/9  (55.6%) | 3/5  (60.0%) | 2/6  (33.3%) | 5/20  (25.0%) | 8/21  (38.1%) |
| ANI started for other manifestations | 0/26 | 5/23  (21.7%) | 7/23  (30.4%) | 2/11  (18.2%) | 0/3 | 0/1 | 9/26  (34.6%) | 9/26  (34.6%) |
| p value*  Partial hematologic response overall | -  - | -  17/43  (39.5%) | -  11/37  (29.7%) | -  5/20  (25.0%) | -  0/9  (0.0%) | -  0/7  (0.0%) | 0.53  - | 1.00  - |

Values are expressed as n/N (%). Values at 3, 6, 12, 18 and 24 months refer to scheduled visits among evaluable patients. “Within 6 months” and “Any time” indicate cumulative complete hematologic response. Denominators reflect patients evaluable for hematologic response at each timepoint based on availability of complete laboratory data

**p values were derived from Fisher exact test comparing patients who initiated anifrolumab for hematologic manifestations versus other indications, considering cumulative CHR within 6 months and CHR at any time*

**Supplementary Table S2** Active organ involvement leading to anifrolumab initiation in patients treated for hematologic manifestations and in those treated for other SLE manifestations

|  | **ANI started for hematologic manifestations (21 patients)** | **ANI started for other manifestations (26 patients)** |
| --- | --- | --- |
| Skin involvement | 6 (28.6%) | 20 (76.9%) |
| Joint involvement | 5 (23.8%) | 15 (57.7%) |
| Serositis | 0 (0.0%) | 1 (3.8%) |
| Neuropsychiatric SLE | 1 (4.8%) | 2 (7.7%) |
| Glomerulonephritis | 0 (0.0%) | 1 (3.8%) |
| Constitutional symptoms | 1 (4.8%) | 0 (0.0%) |
| Gastrointestinal | 1 (4.8%) | 0 (0.0%) |
| Hematologic only | 10 (46.6%) | - |

**Supplementary Table S3.** Comparison of baseline features in patients who achieved *versus* did not achieve complete hematologic response (CHR) within 6 months of anifrolumab initiation

|  | **Responders**  **(n=14)** | **Non responders (n=32)** | **p-value*** |
| --- | --- | --- | --- |
| Age, years | 48.8 (44.9 – 52.8) | 43.8 (34.6 – 51.3) | 0.188 |
| Hematologic involvement as reason for anifrolumab beginning (ref. other involvement) | 5 (35.7%) | 15 (46.9%) | 0.535 |
| Manifestations at baseline |  |  |  |
| Constitutional | 6 (42.9%) | 14 (43.8%) | 1.000 |
| Mucocutaneous | 11 (78.6%) | 19 (59.4%) | 0.316 |
| Joint | 7 (50.0%) | 19 (59.4%) | 0.748 |
| Type of hematologic involvement |  |  |  |
| Lymphocytes <1000 cells/mm³ | 7 (59.0%) | 6/29 (20.7%) | 0.077 |
| Haemoglobin <12 g/dL | 10 (71.4%) | 13 (40.6%) | 0.108 |
| Leukocytes <4000 cells/mm³ | 9 (64.3%) | 19 (59.4%) | 1.000 |
| Platelets <150000 cells/mm³ | 10 (71.4%) | 22 (68.8%) | 1.000 |
| CRP (mg/L) | 1.13 (0.6 – 4.2) | 3.5 (1.9 – 6.7) | **0.008** |
| C3 (mg/dL) | 72 (64 – 96) | 82 (69 – 95) | 0.347 |
| C4 (mg/dL) | 8 (6 – 15) | 12 (8 – 19) | 0.173 |
| Autoantibody profile |  |  |  |
| Anti-dsDNA | 9 (64.3%) | 20/31 (64.5%) | 1.000 |
| Anti-Sm | 3/13 (23.1%) | 12/28 (42.9%) | 0.305 |
| Anti-cardiolipin | 2/13 (15.4%) | 3/25 (12.0%) | 1.000 |
| Anti-B2GP1 | 2/13 (15.4%) | 3/25 (12.0%) | 1.000 |
| Concomitant therapies at anifrolumab beginning |  |  |  |
| Corticosteroids | 7 (50.0%) | 28 (87.5%) | **0.010** |
| Prednisone dosage, mg/day** | 2.5 (0-10) | 5 (4-10) | 0.242 |
| Hydroxychloroquine | 11 (78.6%) | 23 (71.9%) | 0.729 |
| Methotrexate | 4 (28.6%) | 4 (12.5%) | 0.222 |
| Mycophenolate | 3 (21.4%) | 6 (18.8%) | 1.000 |
| Azathioprine | 2 (14.3%) | 5 (15.6%) | 1.000 |
| Rituximab | 0 | 1 (3.1%) | - |
| Belimumab | 1 (7.1%) | 2 (6.3%) | 1.000 |
| IVIg | 0 | 1 (3.1%) | - |

**p values were derived from Fisher exact test for categorical variables and from exact Mann–Whitney test for continuous variables*

**Supplementary Table S4.** Retention rate and adverse events during anifrolumab treatment

| **Discontinuations** (n, %) | **N (% out of 47)**  12 (25.5) |
| --- | --- |
| **Median time to discontinuation,** months (IQR) | 6 (IQR 3.5-6.5) |
| **Causes of therapy discontinuation** (n, %)  Adverse events*  Infections  Inefficacy  Other** | 2/12 (16.7)  3/12 (25.0)  4/12 (33.3)  3/12 (25.0) |
| **Adverse events not leading to discontinuation,** n (%)  Infections  Other*** | 16 (34.0)  14 (29.8)  2 (4.3) |

* 1 liver toxicity and 1 urticaria

** 1 patient request, 1 unplanned pregnancy, 1 planned pregnancy

***1 stenosis of the superior mesenteric artery and the celiac trunk and ulcerated sub-stenosis of the transverse colon, 1 urticaria

**Supplementary Table S5.** Background therapy at baseline and at last follow-up

| **Drug** | **Baseline**  **(n=47)** | **Last follow up**  **(n=47)** |
| --- | --- | --- |
| Prednisone use (n, %)  Median daily dose mg/die (IQR) | 36 (76.6)  5 (2.5-10) | 31 (66.0)  2.5 (0-5) |
| Hydroxychloroquine (n, %) | 35 (74.5) | 32 (68.1) |
| Methotrexate (n, %) | 8 (17.0) | 6 (12.8) |
| Mycophenolate Mofetil (n, %) | 9 (19.1) | 9 (19.1) |
| Azathioprine (n, %) | 8 (17.0) | 3 (6.4%) |
| Cyclophosphamide | 0 (0) | 0 |
| Rituximab | 1 (2.1) | 0 |
| Belimumab | 3 (6.4) | 0 |
| IvIg | 1 (2.1) | 1 (2.1) |
| Other immunosuppressants* | 8 (17.0) | 6 (12.8) |

* *Dapsone, cyclosporine A, tacrolimus, leflunomide*

**Supplementary Figure S1** Longitudinal changes in hematologic parameters during ANI treatment restricted to patients with abnormal baseline values for the given hematologic parameter

Individual patient values and median (IQR) are shown at each scheduled visit. Statistical comparisons refer to changes from baseline at 3 and 6 months of follow-up. Dotted line in each graph indicates the lower limit of normal range
